# Lung Surfactant Deficiency in Severe Respiratory Failure: A Potential Biomarker for Clinical Assessment

**DOI:** 10.3390/diagnostics15070847

**Published:** 2025-03-26

**Authors:** Peter Schousboe, Bülent Uslu, Amalie Schousboe, Lars Nebrich, Lothar Wiese, Henrik Verder, Nikolaos Scoutaris, Povl Verder, Henning Bay Nielsen

**Affiliations:** 1 Department of Pediatrics, Holbæk Hospital, 4300 Holbæk, Denmark; 2 Department of Anesthesia and Intensive Care, Zealand University Hospital, 4000 Roskilde, Denmarkhennin@regionsjaelland.dk (H.B.N.); 3 Department of Infectious Diseases, Zealand University Hospital, 4000 Roskilde, Denmark; 4 Department of Anesthesia and Intensive Care, Zealand University Hospital, 4600 Koege, Denmark; 5 Department of Clinical Medicine, University of Copenhagen, 1172 København, Denmark

**Keywords:** ARDS, bronchial aspirate, surfactant

## Abstract

**Background/Objectives:** Critical lung infection affects alveolar cells and probably also their ability to perform surfactant procedures, but bedside tools for monitoring lung surfactants are lacking. In this descriptive exploratory study, we aimed to evaluate lung surfactant levels in bronchial aspirate (BA) from patients admitted to the intensive care unit due to severe respiratory failure. **Methods:** Bronchial aspirates were collected from nine patients (median age: 72 years, range: 52–85) who required orotracheal intubation. Samples were obtained within 24 h of mechanical ventilation initiation (T1), after three days on a ventilator (T2), and on day seven (T3) for four patients. The concentration of dipalmitoylphosphatidylcholine (DPPC), a key surfactant component, was assessed in the lamellar body precipitate. **Results:** Across the nine patients at T1, the DPPC level was 12 µM (range: 3–20 µM). By T2, the DPPC level declined to 8 µM (range: 2–22 µM), with a statistically significant decrease from T1 (*p* = 0.0039). At T3, the DPPC level in four patients ranged from 2 to 5 µM, though the difference from T2 was not statistically significant. A surfactant biomarker would assist clinical decision-making when dealing with patients in severe respiratory failure where exogenous surfactant therapy may be considered. **Conclusions:** DPPC levels obtained from bronchial aspirate can be measured in patients with severe respiratory failure and may serve as a useful biomarker for lung surfactant status, which suggests the potential for bedside assessment in clinical practice with a dedicated test device.

## 1. Introduction

Lamellar bodies are vesicles released by exocytosis from ATII cells, serving as storage organelles for lung surfactant. Surfactant components are composed of approximately 90% lipids and 10% proteins, with dipalmitoylphosphatidylcholine (DPPC) being the predominant phospholipid [1]. In the alveolar space, the surfactant undergoes transformation into a structure known as tubular myelin, a complex surfactant mesh structure, reducing surface tension, which prevents alveolar collapse during expiration [2,3,4]. Pulmonary infection compromises the integrity of alveolar type II (ATII) cells, potentially impairing their ability to produce surfactants, a key factor in maintaining alveolar stability and facilitating gas exchange [5,6,7]. Critical lung infection may lead to respiratory failure with the development of adult respiratory distress syndrome (ARDS). The pathophysiology of ARDS involves various mechanisms including inflammation, cellular damage, and altered surfactant protein expression. In ARDS patients, surfactant turnover is increased and the concentration is reduced [7]. The use of invasive ventilatory support in severe respiratory failure compromises the integrity of ATII cells [8]. In animal models of induced injuries resembling ventilation-induced lung injury (VILI), the harmful effects of mechanical ventilation can be explored. Such studies have offered valuable insights into the damaging impact of mechanical ventilation alone and in the presence of pre-existing injury [9,10].

The preservation of lung surfactants seems to be important for lung integrity, and in neonates, exogenous surfactant administration is an accepted therapy [11]. In contrast, for individuals with ARDS, there is insufficient evidence to determine whether the administration of exogenous surfactants impacts mortality, the duration of mechanical ventilation, or the number of ventilator-free days [12]. Furthermore, the nebulization of surfactants can be associated with side effects [13]. It is suggested that surfactant aerosol therapy through vibrating-mesh nebulizers could be a viable rescue therapy in adults with severe respiratory failure [14]. Thus, knowledge of the actual lung surfactants could help us to target those patients in need of treatment. DPPC could be a potential biomarker to be used in clinical care to target patients with low surfactants. In a previous study, DPPC levels were reported in bronchial alveolar lavage (BAL) in patients with ARDS [15]. As BAL is an invasive procedure, particularly in patients with severe respiratory failure, bronchial aspirate (BA) may serve as an attractive alternative for the assessment of DPPC. Moreover, BAL involves the liquid flushing of lung compartments of interest [16], while the suction of bronchial aspirate (BA) using a suction catheter inserted into the tracheal tube is simpler. Thus, the collection of BA from intubated patients requires minimal training, does not necessitate the use of a bronchoscope that is controlled by doctors, and can be performed by nurses or medical students as part of their daily routines. Furthermore, compared to BAL, BA has the distinct advantage that it is suitable for patients in ARDS, whereas BAL appears less feasible. The broader perspective is to equip clinicians with a bedside tool for routine surfactant assessment in patients experiencing severe respiratory failure. Monitoring surfactant levels may also be useful when assessing patient progress during weaning from mechanical ventilation.

Here, we hypothesize that DPPC is measurable in BA sampled during daily routine in patients admitted to the intensive care unit (ICU) due to respiratory failure.

## 2. Materials and Methods

In a prospective exploratory trial design, nine patients were considered eligible. These patients were admitted to the ICU for the treatment of ARDS that needed mechanical ventilation, as defined by the most recent Berlin Criteria [17] and international guidelines [18]. This study was approved by the Regional Ethics Committee Region Zealand (VEK SJ-859). Written informed consent was obtained from the patients when feasible or by next of kin as supervised by a guardian doctor. The inclusion criteria were respiratory failure in need of mechanical ventilation at ICU and the fact that BA was obtained after orotracheal intubation being established. Importantly, we wanted consecutive BA sampling preferably separated by at least three days. The exclusion criteria were severe kidney disease requiring dialysis, severe liver failure, known genetic surfactant disorders, an inability to sample BA for any reason, and anticipated transfer to another hospital within 72 h. The exploratory designed study was planned to obtain BA from intubated patients in mechanical ventilation. Here, we present data from patients in whom consecutive sampling was possible.

The clinical practice of ARDS adhered to international guidelines [19], and local recommendations respecting individual adjustments usually are required in clinical practice. ARDS severity was calculated as the ratio between partial arterial O_2_ pressure and the inspired O_2_ fraction (PaO_2_/FiO_2_). PaO_2_/FiO_2_ values below or equal to 100 mmHg (≤100 mmHg) reflect severe ARDS, a score between 100 and 200 mmHg (100 < PaO_2_/FiO_2_ ≤ 200) reflects moderate ARDS, and ratio values ranging above 200 mm Hg but equal to or below 300 mmHg (200 < PaO_2_/FiO_2_ ≤ 300) reflects mild ARDS. Patient characteristics and medical data were obtained from patient medical records.

The aim was to obtain the first sample as soon as possible after orotracheal intubation was initiated and preferably within 24 h after mechanical ventilation was established (T1). A second BA sample was obtained after three days in invasive ventilatory treatment (T2). If possible, BA was aimed to be collected on a third day (T3). Preferably, BA samples were obtained in daytime hours between 9 a.m. and 3 p.m.

Fourier Transform Infrared (FTIR) spectroscopy was used to measure the infrared absorption from material obtained from the bronchi. By passing an IR beam through a dried sample of bronchial aspirate, it provided insights into the molecular vibrations, aiding the identification of chemical bonds and functional groups within the material. Following the inclusion of the patient, the designated intensive care nurse used a suction catheter to collect the BA directly into a 12 mL polypropylene tube attached to the plastic tubing, ensuring no risk of contamination. The BA material, typically 1–1.5 mL, collected for the analysis of DPPC was stored in a refrigerator (max +5 °C), typically overnight [20]. The BA sample (100 µL) was pre-treated with 100 µL of tris-2-carboxyethyl-phosphine (20 mM) in saline (Thermo Scientific, H51864.AA, Waltham, MA, USA) to reduce protein disulphide bridges and, in this way, dissolve protein clusters. To omit the dilution of the samples by the added TCEP, 200 µL of the mixture was further processed. Firstly, a minor centrifugation step was performed to achieve a cell- and particle-free supernatant, and secondly, a final centrifugation step was carried out at 4000× *g* for 4 min. This resulted in a lamellar body precipitate, consistent with the initial sample volume, which was dried on a CaF2 disk (Crystran, Poole, UK, CAFP13-0.5) and scanned by infrared spectroscopy. The resulting spectrum was then analyzed by a mathematical algorithm trained on reference samples, verified by the mass spectrometry of DPPC.

BA samples were processed by a Sime Diagnostics Alpha+ device (London, UK) that uses mid-infrared spectroscopy to provide spectra reflecting the presence of DPPC. The final infrared spectra of BA were analyzed by principal component analysis [20].

Data are presented as median and ranges. Statistical analyses were conducted by non-parametric statistical methods (Wilcoxon signed rank test). *p* < 0.05 indicated a statistically significant difference. Algorithm statistical analysis was performed by R software (version 4.0.3/4.0.5).

## 3. Results

We present data from nine patients with ARDS (median age: 72 years; range: 52–85) who underwent consecutive bronchial aspirate (BA) sampling. Patient demographics are detailed in Table 1A, while Table 1B outlines the comorbidities accompanying the ARDS diagnosis. Two patients were diagnosed with COVID-19 due to SARS-CoV-2 infection and, therefore, presented COVID-19, whereas the remaining seven experienced respiratory failure from non-COVID-19 causes. All patients were admitted to the ICU for the treatment of severe respiratory failure requiring mechanical ventilation, as reflected in the ventilatory settings during the initial phase of treatment (Table 2A). Notably, approximately half of the patients were experiencing severe respiratory failure and required an inspired oxygen fraction (FiO_2_) above 0.5 to maintain adequate respiratory support.

Thus, three patients had severe ARDS, five had moderate ARDS, and one had mild ARDS. This condition necessitated sedation, as indicated by the RAS score (Table 2B). The patients stayed in the ICU (median 4, range 3–27 days) of which at least three days were under mechanical ventilation. Six patients had a fatal outcome, and three patients survived. The initial DPPC concentration is shown in Table 2B, along with the outcome and sedation conditions.

**Figure 1:**
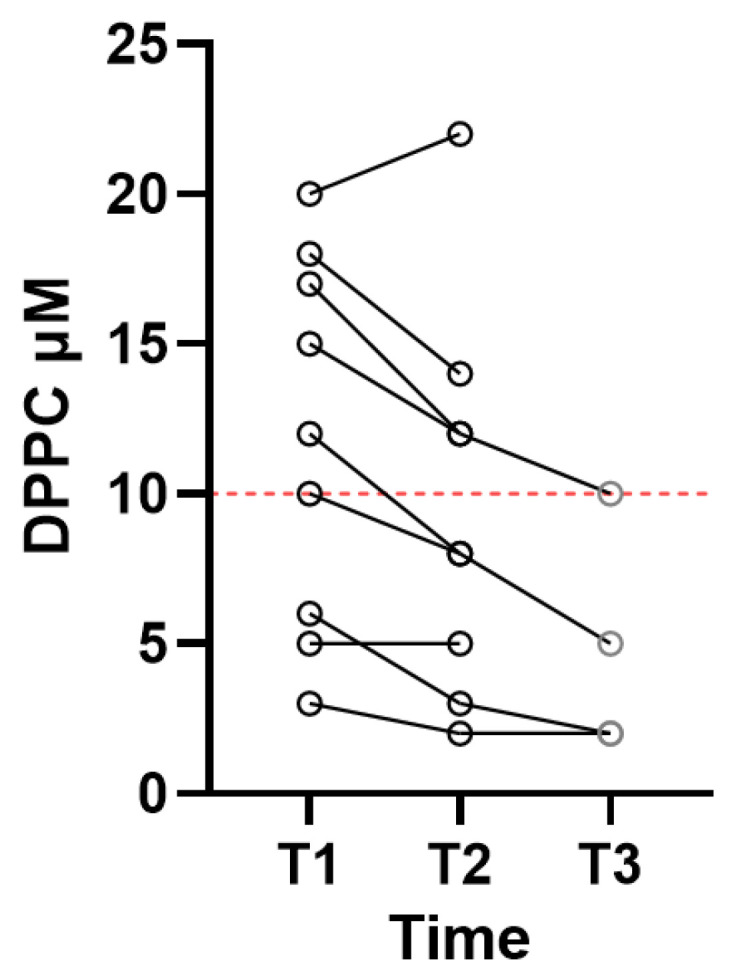
Individual DPPC levels in patients with adult respiratory distress syndrome. Samples were obtained within 24 h after mechanical ventilation was established (T1, *N* = 9), following three days on a ventilator (T2, *N* = 9), and for four patients, samples were also obtained on day 7 (T3, *N* = 4). The red stipulated line indicates a critical DPPC level.

BA sampling was performed on T1 and T2, while a third BA sample (T3) was obtained following approx. 7 days of ventilatory therapy in four patients. Figure 1 shows the individual levels of DPPC where patients were followed from T1–T2, and in four patients, BA was also obtained on T3. On T1, the DPPC median level was 12 µM (range 3–20 µM) for the nine patients. On T2, the measurements showed a median DPPC level of 8 µM (range of 2–22 µM). Analysis of the surfactants in patients with T1 and T2 samples showed a significant difference, with *p* = 0.0039, suggesting a decline in the level of surfactants in patients with severe respiratory failure during treatment at the ICU. In the four patients in whom BA was obtained on T3, the level of DPPC ranged from 2 to 5 µM, with a median value of 3.5, without a statistically significant difference from the DPPC levels at T2.

To assess the impacts of outcomes on surfactant levels, we aimed to analyze the data separately. Among the six patients with a fatal outcome, the median of the initial DPPC measurement was 9 µM (range 3–18 µM). Although the statistical power was insufficient when compared to the DPPC levels in the three patients (DPPC at 10, 17, and 20 µM) with better outcomes, it points to a dynamic variation in the surfactant, which could predict an outcome. In support of this observation is the fact that in four patients in whom BA was obtained at T3, the level of DPPC ranged from 2 to 5 µM (T2–T3 *p* = 0.112); all patients in this group had fatal outcomes. Notably, the COVID-19 patients had surfactant levels below 10 µM, suggesting a cut-off for respiratory integrity. While low surfactant levels indicate a life-threatening risk, some patients with fatal outcomes exhibited higher surfactant levels, suggesting that other factors may play more significant roles.

## 4. Discussion

This exploratory study evaluated lung surfactant levels by measuring DPPC in broncho-aspirate (BA) samples from ARDS patients. The key findings are that (i) BA sampling is feasible in the routine care of patients with severe respiratory failure, (ii) DPPC is measurable in BA, and (iii) data may point to an association between low DPPC and outcome. Notably, in patients undergoing repeated sampling, the data suggest that BA collection remains viable even in complex and dynamic clinical settings.

### 4.1. Implications

The observed DPPC levels, indicative of the presence of surfactant, align with previously reported data from ARDS patients whose lung fluid was obtained via BAL [15]. Thus, in patients with severe respiratory failure, surfactants are measurable in lung fluid obtained by BA, suggesting further elaborations of its relevance in clinical practice. Observations also indicate that low surfactant levels, as reflected by DPPC levels, could serve as a prognostic marker in patients undergoing invasive ventilatory therapy.

The role of surfactants in ARDS has been investigated through the exogenous administration of surfactants to patients. However, a meta-analysis concludes that this treatment approach did not improve mortality or oxygenation in ARDS patients [21]. Also, oxygenation failed to improve in patients with severe COVID-19 due to a high turnover of surfactants [22]. Reviews of clinical trials indicated that while surfactant therapy might improve oxygenation, it did not significantly impact long-term survival rates [23,24]. A potential bias may stem from the assumption that exogenous surfactant administration benefits all ARDS patients, which automatically include those with adequate surfactant levels. The lack of a useful method to assess surfactant concentration may have led to conclusions that exogenous surfactants have no significant effect on lung function. Such a viewpoint has been investigated, and it was shown that a subgroup of ARDS patients with severe cases due to pneumonia or aspiration who received surfactant treatment showed significantly improved oxygenation and increased survival [25]. Our findings show significant variability in surfactant levels among ARDS patients, with some having seemingly adequate levels but with fatal outcomes. This also indicates that factors other than surfactant deficiency may contribute to ARDS.

Surfactant administration may be advantageous for patients with suppressed endogenous surfactant levels, such as acute lung injury (ALI), where compromised ATII cells reduce surfactant production, negatively impacting gas exchange, which is frequent in direct ARDS phenotypes. Additionally, it is important to consider that mechanical ventilation can lead to ventilator-induced lung injury (VILI) [9,26,27], which can enhance the injury caused to an already ALI compromised lung [28] with surfactant deficiency. It is possible that the ventilatory mode in our analysis influenced the integrity of surfactant production by ATII cells. While we assessed surfactant levels in BA, we could not determine whether the variations were due to VILI, direct effects on ATII cells, or inadequate ARDS treatment. Additionally, infections may contribute to this process by degrading phospholipids and surfactant proteins. However, since we isolate lamellar bodies and, in this way, measure DPPC content before its release into the alveoli [20], our findings suggest that the process of lamellar body exocytosis from ATII cells may be impaired. This would also impact the administration of exogenous surfactant, as the primary need is to enhance the endogenous production of surfactant. In adults, the administered surfactants have a high turnover and only a temporary effect on oxygenation [7,22,29]. However, the long-term effects on endogenous surfactant production remain uncertain [30].

Previous studies have suggested that the early administration of surfactants could provide potential benefits [31]. BA or potentially tracheal aspirates collected before intubation could serve as valuable sources for assessing surfactant levels and aid in decision-making [31]. When endogenous surfactant production is already low, there is only sporadic evidence that supplemented surfactant stimulates an increase in endogenous production in adult humans. This contrasts with preterm infants, where surfactant therapy is a standard treatment for neonatal respiratory distress syndrome and supplemented surfactants allow the lungs time to establish sufficient surfactant production. The method could be used to assess the de novo synthesis of surfactants in treated patients, which appears to be crucial when the goal is to restore a balanced lung surfactant, as well as to further investigate for which ARDS groups this could be relevant. In experiments with adult mice, it was shown that de novo synthesis of surfactants took place [32].

Before initiating mechanical ventilation, a quick clinical method for assessing surfactant levels in ARDS patients could involve evaluating surfactant concentration in tracheal aspirates collected through nasal access to the trachea. Measuring surfactant levels in lung fluid obtained through tracheal suction, BA, or BAL could improve clinical trials by evaluating whether exogenous surfactant administration in ARDS patients enhances the production of endogenous surfactants through lamellar bodies. Another advantage of measuring lung surfactant levels is the ability to monitor surfactant production, aiding in determining the optimal time for extubation once sufficient levels are achieved. The assessment of surfactants by the sampling of BA for the analysis of DPPC could prove to be a valuable technique in clinical practice. The current method has been developed into a bedside monitoring tool that provides an easily applicable evaluation of surfactant biomarker status. This allows for assessing the status of the biochemically necessary fundament for alveoli integrity that, in turn, would determine when an intubated patient could leave mechanical ventilatory support. Additionally, a surfactant biomarker would aid clinical decision-making for patients with severe respiratory failure, helping to determine when exogenous surfactant therapy may be necessary. While we utilized infrared spectroscopy and mathematical algorithms to assess surfactant levels, other studies have employed mass spectrometry to analyze surfactants in BAL fluid from ARDS patients. Although mass spectrometry offers detailed insights, its complexity and time-consuming nature may limit its practicality for evaluating surfactant dynamics. In contrast, infrared spectroscopy is more user-friendly and is currently being developed for bedside surfactant measurements [20].

### 4.2. Limitations

The data should be interpreted with caution, considering the size of the dataset. Although the dataset is small, it suggests that a significant decrease in DPPC levels during mechanical ventilation may be associated with a poor outcome. In particular, for patients with low levels of DPPC, it is noteworthy that none survived. Since the airway suction routine through the tracheal tube was performed by various ICU nurses during their shifts, and although they adhered to the department’s standard care guidelines, variability in the sampling process of BA is difficult to exclude. Such variability could potentially have an influence on the quality of data. However, given the small volume used in the test relative to the total volume of the collected samples, any variation in the samples is considered low. In addition, during the sampling process, it was a goal to minimize any potential dilutional effect.

In addition to the mentioned limitations, this study is primarily constrained by its exploratory design, the small patient cohort studied, our limited knowledge regarding specific sample collection, and the data outcomes. Consecutive surfactant collection assessed by a point-of-care device has, to the best of our knowledge, not been performed before and needs to be evaluated carefully to prepare for the next study, where we will focus on a swift and easy way to access lung surfactants before admission to mechanical ventilation. For a firm conclusion on any association between low surfactants and bad outcomes for ICU patients, large-scale studies with outcome as the primary factor need to be performed.

## 5. Conclusions

In patients with severe respiratory failure, lung surfactants are quantifiable as the DPPC biomarker, suggesting the need for further elaborations of its relevance in clinical practice.

Future research is needed to advance the assessment of DPPC and, in turn, surfactants in lung mucous for routine clinical use. Such research will need additional studies to confirm the validity and sampling process of the methods. In addition, the dynamics of DPPC levels over time in intubated patients would add to the understanding of when “normal” surfactant levels are achieved once respiratory failure has cleared up. Third, the influence of different settings of ventilatory support on surfactant integrity could also be explored.

## Figures and Tables

**Table 1:** (**A**) The characteristics of patients with acute respiratory distress syndrome (ARDS). (**B**) The occurrence of various comorbidities in patients with acute respiratory distress syndrome (ARDS).

(**A**)				
**ID#**	**Age yrs**	**Gender**	**Height cm**	**Weight kg**
1	62	F	173	101
2	70	M	178	82
3	74	F	165	101
4	85	M	172	76
5	52	M	174	90
6	76	M	180	61
7	60	F	174	113
8	72	F	158	79
9	76	F	166	60
(**B**)				
**ID#**	**Co-Morbidities**			
1	COVID-19, COPD, hyperthyroidism			
2	COVID-19			
3	GBD, cancer, obesity, hypertension			
4	Cerebral stroke, hypertension			
5	Diabetes, hypertension			
6	COPD, diabetes, PAD			
7	AML, cancer, obesity, psoriasis			
8	CVD, COPD, diabetes, hypertension, benzodiazepine intoxication			
9	GD, CVD, lung cancer, hypertension			

ID#, the patient identification number; F, female, M, male; kg, kilogram. AML, acute myeloid leukemia; COPD, chronical obstructive lung disease; COVID-19, coronavirus disease 2019; CVD, chronical vascular disease; GD, Graves’ disease; GBD, Guillain–Barre disease; PAD, peripheral artery disease, yrs. years.

**Table 2:** (**A**) Mechanical ventilation settings for patients with acute respiratory distress syndrome (ARDS). (**B**) Sedation scores and outcomes in patients with acute respiratory distress syndrome (ARDS).

(**A**)					
**ID#**	**ARDS Score mm Hg**	**TV (mL/kg)**	**FiO_2_ (%)**	**PEEP cm H_2_O**	**MV (Days)**
1	159	8.6	60	8	25
2	88	6.8	100	10	17
3	149	6.8	40	8	4
4	250	6	40	5	3
5	107	7.8	50	10	16
6	273	8.2	25	12	4
7	266	6.1	30	3	3
8	150	7.5	60	5	4
9	119	6.1	65	8	27
(**B**)					
**#**	**RAS Score**	**ICU (Days)**	**Outcome**	**Initial DPPC (µM)**	
1	−1	25	†	3	
2	−4	17	†	3	
3	−1	16	O	10	
4	−1	3	†	6	
5	−1	32	O	17	
6	−1	28	†	12	
7	−4	14	†	18	
8	−1	12	O	20	
9	−1	27	†	15	

Individual respiratory variables for ARDS patients (ID#). ARDS score, ratio between arterial O_2_ tension, and inspired O_2_ fraction (FiO_2_) with values < 100 mmHg indicating severe ARDS, 100 ≤ PaO_2_/FiO_2_ ≤ 200 indicating moderate ARDS, and 200 < PaO_2_/FiO_2_ ≤ 300 indicating mild ARDS; MV, number of days under mechanical ventilation; PEEP, positive end expiratory pressure; TV, tidal volume. RAS is the sedation score. Outcome shows whether patients had a fatal (†) or good (O) fate. DPPC is the dipalmitoylphosphatidylcholine level at the time of inclusion.

## Data Availability

No further data than those present in the manuscript are available.
